# Isolation and next generation sequencing of archival formalin‐fixed DNA

**DOI:** 10.1111/joa.13209

**Published:** 2020-05-19

**Authors:** Ahlam Alqahtani, Andrew Skelton, Lorraine Eley, Srinivas Annavarapu, Deborah J. Henderson, Bill Chaudhry

**Affiliations:** ^1^ Bioscience Institute Faculty of Medical Sciences Newcastle University Newcastle upon Tyne UK; ^2^ Bioinformatic Support Unit Faculty of Medical Sciences Newcastle University Newcastle upon Tyne UK; ^3^ Department of Cellular Pathology Newcastle Hospitals NHS Foundation Trust Newcastle upon Tyne UK

**Keywords:** archival DNA, Chelex 100, formalin, next generation sequencing, Uracil‐N‐glycosylase

## Abstract

DNA from archived organs is presumed unsuitable for genomic studies because of excessive formalin‐fixation. As next generation sequencing (NGS) requires short DNA fragments, and Uracil‐N‐glycosylase (UNG) can be used to overcome deamination, there has been renewed interest in the possibility of genomic studies using these collections. We describe a novel method of DNA extraction capable of providing PCR amplicons of at least 400 bp length from such excessively formalin‐fixed human tissues. When compared with a leading commercial formalin‐fixed DNA extraction kit, our method produced greater yields of DNA and reduced sequence variations. Analysis of PCR products using bacterial sub‐cloning and Sanger sequencing from UNG‐treated DNA unexpectedly revealed increased sequence variations, compared with untreated samples. Finally, whole exome NGS was performed on a myocardial sample fixed in formalin for 2 years and compared with lymphocyte‐derived DNA (as a gold standard) from the same patient. Despite the reduction in the number and quality of reads in the formalin‐fixed DNA, we were able to show that bioinformatic processing by joint calling and variant quality score recalibration (VQSR) increased the sensitivity four‐fold to 56% and doubled specificity to 68% when compared with a standard hard‐filtering approach. Thus, high‐quality DNA can be extracted from excessively formalin‐fixed tissues and bioinformatic processing can optimise sensitivity and specificity of results. Sequencing of several sub‐cloned amplicons is an important methodological step in assessing DNA quality.

## Graphical abstract

Formalin fixation is routinely used for storage and histological processing of human tissue samples. However, formalin fixation prevents effective PCR and sequencing. To overcome this we developed a novel DNA extraction process which generates DNA capable of producing longer amplicons. However, we show that corrupted bases still exist, and that treatment with uracil‐N‐glycosylase does not enhance identification of normal sequence. Using formalin fixed tissue and freshly isolated DNA from the same individual we carried out. Next Generation Exome Sequencing and found that although formalin‐fixed DNA performed less well during sequencing, using bioinformatic filtering it was possible to obtain moderately high sensitivity and specificity of base reads. Thus, although formalin fixed DNA should not be used as a primary source for variant discovery, it may be used to confirm variants.

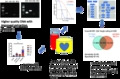

## 1 INTRODUCTION

Formalin provides convenient fixation and storage of human tissue samples prior to routine pathological investigation (Fox *et al.,* 1985). It maintains tissue integrity, allows application of a range of histological stains and permits immunohistochemistry, often enhanced through antigen retrieval (Shi *et al.,* 1991). Genetic analyses are increasingly being used to diagnose human disease. For example, somatic mutations found in tumour biopsies are of prognostic importance and can be used to guide therapy in many cancers (Dancey *et al.,* 2012). Unravelling the genetic basis of inherited disease is also reliant on DNA, and historical formalin‐fixed (FF) or formalin‐fixed paraffin wax‐embedded (FFPE) samples may be the only source of DNA from affected but deceased family members. However, although only minimal fixation in formalin (preferably none) is suggested for tissues that might be used for genomic studies, in practice the samples may have been fixed for weeks or months before embedding in paraffin wax. Moreover, there are many archives of congenitally malformed human hearts (and other organs) used for educational and anatomical research purposes (Crucean *et al.,* 2017). As the majority of genes causing congenital heart malformation still remain to be discovered, it is possible that these accurately phenotyped hearts, permanently stored in formalin to preserve integrity of the specimen, could provide a valuable resource to identify gene variants and to correlate causative mutations with morphological appearances.

FF tissues are not generally considered suitable for genetic/genomic analyses due to a range of corrupting effects directly attributed to formalin. One significant issue is the formation of methylene bridges between proteins and DNA (Metz *et al.,* 2004). However, these are potentially reversible (Hoffman *et al.,* 2015) and in recently developed techniques such as chromatin immunoprecipitation (ChIP), formalin is specifically used to bond DNA fragments to relevant peptides and allow their isolation for sequencing (Nelson *et al.,* 2006a). Formalin exposure also produces deamination of cytosine to uracil (Williams *et al.,* 1999). Such deamination events occur during life, but Uracil‐N‐glycosylase (UNG) normally excises the deaminated base, allowing DNA repair mechanisms to replace the missing base with reference to the complementary strand (Do, 2012). UNG is commercially available and has been suggested as a treatment for DNA extracted from FF material (Do, 2012). Historically, the major limitation to sequencing FF material was due to extensive fragmentation of DNA (Suzuki *et al.,* 1994), which prevents chain termination (Sanger) sequencing. However, next generation sequencing (NGS) technologies perform best with short‐length template and shearing or transposon‐mediated cleavage to produce efficiently fragments of 150–250 bp, produces paired‐end reads of 75 bases in length (Head *et al.,* 2014).

Following our recent morphological study of congenitally malformed hearts (Crucean *et al.,* 2017) we were asked by clinicians and anatomists whether this extensive collection of fully morphologically characterised organs, routinely stored in formalin, could be used to discover causative mutations by exome sequencing. In the absence of studies dealing with genomic interrogation of heavily formalin‐fixed samples, we addressed ourselves to this clinically important question. We first sought to isolate high quality genomic DNA that would be suitable for both NGS and Sanger sequencing. Although successful isolation of DNA can be assessed in terms of yield and overall fragment length, for our studies we were more concerned with the efficacy of the extracted DNA to act as a template within the PCR reaction and the accuracy of sequence obtained. As such, this study is the first to analyse systematically the distribution of errors in PCR amplicons through bacterial sub‐cloning of transcripts. We also assessed the effect of enzymatic treatment with UNG and, surprisingly, found that a different range of artefacts are present in PCR amplicons after UNG is employed. Finally, we performed next generation exome sequencing on myocardial tissue that had been stored for 2 years in formalin and compared the results with those obtained from NGS of DNA freshly extracted from peripheral blood from the same patient. Although the high rates of false‐positive results in FF‐DNA preclude its use as a primary source of DNA for discovery of unknown causative genes, we suggest that it may be useful as a confirmatory DNA source.

## 2 METHODS

### 2.1 Tissue samples

A single piece of surgically discarded pulmonary artery that had been stored in buffered formalin at room temperature for over 8 months, was used to investigate optimal methods for DNA extraction.

The comparison of genomic sequence by NGS used DNA extracted from a small biopsy of right ventricular myocardium taken from a heart that had been stored in buffered formalin for 2 years (following transplant operation). DNA obtained from peripheral blood lymphocytes was available from the same individual. In both cases, material was obtained from the Department of Cellular Pathology, Royal Victoria Infirmary, Newcastle upon Tyne, and studies were carried out under the approval of the Newcastle and North Tyneside 1, NRES Committee; REC Reference: 15/NE/0311.

### 2.2 Tissue processing and digestion

Pieces of the pulmonary artery sample were cut into approximately 1‐mm^3^ cubes, weighed and washed in glycine‐Tris‐EDTA (GTE) buffer (100 mM glycine, 10 mM Tris‐HCL pH 8.0 and 1 mM EDTA) using a shaker water bath at 37°C with changes of GTE buffer as indicated in experimental results. Tissues were then digested in 3 ml lysis buffer (1 M Tris‐HCl pH 8.0, 10% sodium dodecyl sulphate [SDS], 0.5 M EDTA) supplemented with different quantities of proteinase K (Sigma‐Aldrich) as indicated. Optionally, tissue was treated with 2 U Uracil‐N‐glycosylase (UNG; New England Biolabs) at 50°C for 1 hr (Liu *et al.,* 2013). Chelex resin (Bio‐Rad) was added to make a final concentration of 10% and the resulting solution either heated to 100°C or autoclaved to 120°C and 980 mbar pressure for 10 min. After treatment with 40 µg ml^‐1^ RNaseA (Sigma‐Aldrich) for 5 min at room temperature, DNA was isolated by phenol‐chloroform extraction, resuspended in 50 ml Tris‐EDTA buffer and stored at −20°C (Green and Sambrook, 2012‬).

Extraction of DNA from right ventricular biopsy was performed according to this schedule, under the optimised conditions indicated in experimental results.

DNA was also extracted from the pulmonary artery sample using the GeneRead system (Cat. No. 180,134, Qiagen group, UK) according to the manufacturer’s instructions.

Isolation of DNA from patient blood, i.e. lymphocyte‐derived DNA (LD‐DNA), was performed using the Illustra Nucleon BACC Genomic DNA Extraction Kit (GE Healthcare Life Sciences, RPN‐8502, UK) according to manufacturer’s instructions.

### 2.3 Quantification of DNA

The yield (ng mg^‐1^ original tissue) and purity (260/230 ratio) was assessed using NanoDrop spectrophotometry (Nanodrop‐8000, Thermo Fisher Scientific). Extracted DNA was visualised by 0.8% gel electrophoresis against a 10‐kb ladder (NEB).

### 2.4 PCR efficiency assay

Conventional PCR amplification of genomic gene DNA was performed to generate 200‐, 400‐, 600‐ and 800‐bp‐sized products from the *HAND1* gene. The final PCR reaction volume was 25 μl (2 μl DNA (25 ng μl^‐1^); 1.25 μl of 10 μM of each primer; 0.5 μl of 10 μM of each dNTP; 2 μl of 10 mg ml^‐1^ bovine serum albumin (final concentration. 1 μg μl^‐1^ B); 5 μl of 5× Q5 Reaction Buffer and 5× Q5 High GC Enhancer; and 1 U DNA Taq polymerase (Q5® High‐Fidelity DNA Polymerase, NEB, Ipswich, MA, USA). Thermal cycler conditions were: 94°C for 5 min; 40× (94°C for 1 min; 60°C for 30 s; 72°C for 1 min); 72°C for 10 min. Amplicons were resolved with ethidium bromide on 1% agarose gels. Gels were digitally imaged (GelDoc‐It2 Imager, Labortechnik) and quantification carried out using Fiji (Schindelin *et al.,* 2012).

Primer sequences:

- *HAND1* 200 bp ‐ Fwd GCCCTATCTCCATTTCCCTA, Rev CAGGAAGTGCAGCGACAAAA;
- *HAND1* 400 bp ‐ Fwd CAACCACACACTTGGATCGC, Rev CAGGAAGTGCAGCGACAAAA;
- *HAND1* 600 bp ‐ Fwd GCCCAGTCGAGAAGAGGATT, Rev CAGGAAGTGCAGCGACAAAA;
- *HAND1* 800 bp ‐ Fwd ACCGGTAAGAAGACAGTGGG, Rev ACACAGCCTCCTTCGACTAC.

### 2.5 Bacterial sub‐cloning and Sanger sequencing

To facilitate Sanger sequencing, PCR products were inserted into the pGEMT‐easy vector system II (Thermo Fisher Scientific, Inc.). Twenty colonies were sub‐cloned and amplicons purified using QIAprep Spin Miniprep Kit (Cat. No. 27,104, Qiagen Group) according to the manufacturer’s instructions. Plasmid insert DNA was subjected to Sanger sequencing using both forward SP6 (Cat. No. Q5011, Promega) and reverse T7 (Cat. No. Q5021, Promega) direction primers.

### 2.6 Next generation exome sequencing

Libraries for NGS were produced from Illumina Nextera exome sequencing kits (Illumina). DNA obtained from formalin‐fixed right ventricle was processed using TrueSeq Exome kit (Illumina, San Diego, CA, USA), and lymphocyte‐derived DNA using TrueSeq Rapid Exome kit (Illumina) according to the manufacturer’s instructions. Quantification for NGS was carried out using the Genomic DNA ScreenTape Analysis kit.

tape station analysis was used (2200 tape station system, software version 8.4.1, Agilent). An Illumina NextSeq 500 (Illumina) with equal loading of library preparation was used to collect 80 million paired end reads, 75‐bases in length, divided across four flow cells. Following sequencing, several global analyses were reported by the Illumina platform, including number of reads, read depth and GC content profiles.

### 2.7 Analysis of exome sequencing

Bioinformatic analysis was carried out using GATK 3.4 workflow on a high‐performance computing cluster (Newcastle University). The bioinformatic processing scripts are available at https://github.com/AndrewSkelton/PID‐WES‐GATK3.4‐SGE. Briefly, after reads were pooled and converted to BAM format, they were aligned to the Hg19 genomic scaffold using BWA‐mem. Duplicate reads were identified and realignment considering known insertions and deletions was carried out using Picard Tools. Base quality score recalibration was performed, and HaplotypeCaller used to identify variants. The HaplotypeCaller was used in different modes. For read filtering, that is, applying a series of pre‐defined standard limits normally used for evaluation of individual human exomes (QualByDepth [QD] 2.0; FisherStrand [FS] 60.0; RMSMappingQuality [MQ] 40.0; MappingQualityRankSumTest [MQRankSum] −12.5; ReadPosRankSumTest [ReadPosRankSum] −8.0; StrandOddsRatio [SOR] 3.0), HaplotypeCaller was operated in single sample mode. Joint calling required the use of genomic variant call (gVCF) mode and utilised an exome catalogue (a large series of 216 unrelated exomes). This catalogue provides data to indicate general population baseline statistics and the filtering of sequencing results using this is called ‘variant quality score recalibration’ (VQSR). Analysis of the LD‐DNA and FF‐DNA samples was performed on each separately and also on both at the same time using VQSR. The variants produced were annotated using Ensembl Variant Effect Predictor (VEP version 90) along with LOFTEE and dbnsfp plugins. Datasets were explored and analysed in R.

As default settings, both hard filtering and joint calling analyses use data provided at the time of sequencing to limit analysis based on the quality of bases in the individual reads. This metric is the Phred (Q) score. For example, a score of Q30 describes a base with an error probability of 1 in 1,000, i.e. one that is likely to be 99.9% accurate. We were therefore able to re‐run analyses at lower stringency (Q20: including data likely to be 99.0% accurate; Q10: data likely to be 90% accurate).

### 2.8 Statistics

All experiments were run in triplicate and data presented as an average and standard error (SEM). Statistical significance was evaluated using one‐way ANOVA or unpaired *t* test (SPSS version 22, IBM) Only *p *< .05 was accepted as significant and correction for multiple testing was included.

## 3 RESULTS

### 3.1 Combined autoclaving and Chelex resin improve DNA quality

**Figure 1:**
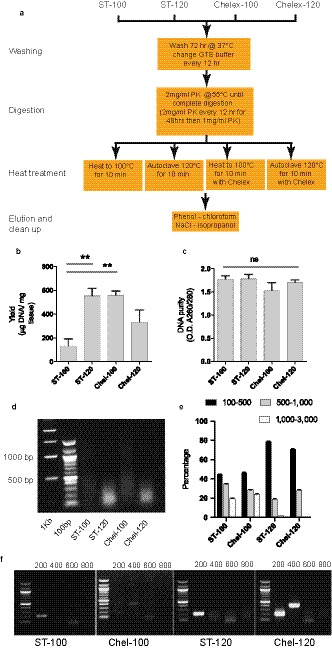
Isolation of high‐quality DNA from excessively formalin‐fixed human tissue. (a) Flow diagram of alternate DNA extraction protocols evaluated. See methods and text for full details. (b) Yield of DNA and (c) purity (Nanodrop). (d) Extracted genomic DNA visualised on 0.8% agarose gel and (e) quantified according to fragment length. (f) Efficacy of DNA extracted as template for PCR primer sets to produce 200‐, 400‐, 600‐ and 800‐base pair (bp) amplicons, 100‐bp ladder. Error bars: mean and SEM. **p < .02. ns = non‐significant. ST‐100 = standard protocol with heating to 100°C, ST‐120 = standard protocol with autoclaving at 120°C, Chel‐100 = protocol incorporating Chelex resin and heating to 100°C, Chel‐120 = protocol incorporating Chelex resin and autoclaving at 120°C. GTE = Glycine‐Tris‐EDTA buffer, PK = proteinase K

Prior to carrying out NGS, we first sought to extract genomic DNA that would produce optimal performance as a template for PCR. A standard method to isolate DNA from tissues is by tissue digestion with proteinase K (PK), followed by heating to 100°C to inactivate the enzyme. After treatment with RNAse, genomic DNA can then be recovered by salt precipitation or with a resin column for downstream applications (Green and Sambrook, 2012‬). Heating to higher temperature has been suggested to improve DNA extraction (Idris, 2015) and we asked whether this, in combination with Chelex resin, might improve the quality of DNA extracted in comparison with previous methods (Legrand *et al.,* 2001; Cao *et al.,* 2003; Shi *et al.,* 2004; Nelson *et al.,* 2006b; Wang *et al.,* 2013; Pandey *et al.,* 2014; Idris, 2015). A single piece of human pulmonary artery stored in formalin for 8 months was used to evaluate different conditions for DNA extraction. Pieces were diced into 1‐mm^3^ cubes and washed extensively in glycine‐containing wash buffer. Digestion with repeated additions of PK (Cao *et al.,* 2003; Shi *et al.,* 2004) was then performed. At this stage, heating to 100°C was compared with autoclaving to 120°C (Shi *et al.,* 2004; Nelson *et al.,* 2006b), with or without Chelex resin (Legrand *et al.,* 2001; Wang *et al.,* 2013; Idris, 2015) (Figure 1a). In comparison to the basic extraction protocol, there was a four‐fold increase in DNA yield when samples were either autoclaved at 120°C, or alternatively Chelex resin was employed (Figure 1b). Surprisingly, when Chelex was combined with autoclaving, the yield was not significantly greater than from heating to 100°C alone (Figure 1b). In all cases, high purity DNA (Nanodrop 280/260 absorption) was obtained (Figure 1c).

Gel electrophoresis and densitometry (Schindelin *et al.,* 2012) was used to demonstrate the distribution of fragment lengths (Figure 1d,e). Heating to 100°C led to isolation of fragments over 1,000 bp in length, but autoclaving led to the loss of these long fragments and greater representation of fragments under 500 bp. Chelex had no additional effect. To examine the efficacy of the extracted DNA within the PCR reaction we designed primer sets to amplify products of 200, 400, 600 and 800 bp length from exon 2 of the *HAND1* gene. The basic method of extraction, heating to 100°C, was only capable of producing amplicons of 200 bp length. Whereas autoclaving produced a greater amount of the 200‐bp PCR product, treatment with Chelex at 100°C produced a small amount of the 400‐bp product. When treatment with Chelex and autoclaving were both combined a significant amount of the 400‐bp PCR amplicon was obtained (Figure 1f), which reflects the maximum length of DNA template fragments obtained (Figure 1d,e).

**Figure 2:**
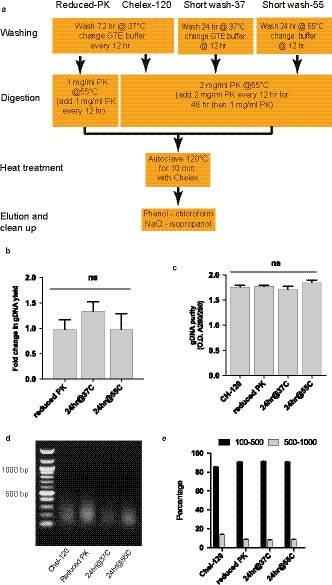
Reduced washing and PK concentration do not affect DNA extraction. (a) Flow diagram indicating reductions of washing and PK to ST‐120 protocol. See Methods for full details. No change in yield (b), purity (c) or fragment length (d,e) of DNA. GTE = Glycine‐Tris‐EDTA buffer, PK = proteinase K; ns = non‐significant

**Figure 3:**
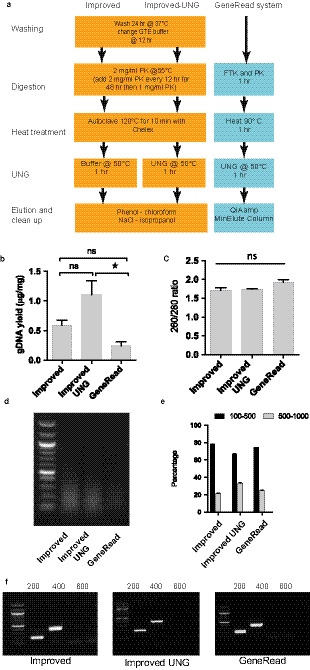
Effect of Uracil‐N‐glycosylase (UNG) and comparison with GeneRead DNA extraction system (Qiagen). (a) Flow diagram indicating incorporation of UNG into improved protocol and comparison with GeneRead system. (b) Greater yield of genomic DNA (gDNA) with improved/UNG method compared with GeneRead system, with no loss of purity (c). Fragment length visualised on gel (d) and (e) quantification by gel densitometry. (f) All methods provided DNA template capable of producing 400‐base pair (bp) amplicons. *p < .05. ns = non‐significant

Having demonstrated the synergistic effect of Chelex resin and autoclaving on the quality of DNA obtained from FF tissue, we next asked whether it might be possible to reduce pre‐washing and the amount of PK used in order to reduce the time required and cost of the procedure. Previous reports have suggested methods for extraction of genomic DNA from FF samples but, empirically, used extensive washing steps (Pandey *et al.,* 2014) and large amounts of PK (Legrand *et al.,* 2001; Pandey *et al.,* 2014). To assess these requirements, we compared extensive washing and PK usage with protocols using less (Figure 2a). Reduction of washing from 72 to 24 hr duration did not affect the yield or quality of DNA obtained. Nor did washing at an increased temperature of 55°C (Legrand *et al.,* 2001), or halving the amount of PK used, alter the yield purity or fragment length distribution of the DNA isolated (Figure 2b–e). On the basis of these refinements, this final ‘improved protocol’ was used to isolate formalin‐fixed DNA for the remaining studies (Figure 3).

### 3.2 UNG improves DNA yield but does not improve the fidelity of amplicon sequence

UNG can be used to overcome the deamination effects of formalin by excising mutated uracil bases from the DNA strand (Do, 2012). Used in vitro*,* this means that corrupted DNA template is severed, favouring intact template. Commercial kits designed for extraction of DNA from formalin‐fixed tissues, for example the GeneRead system (Qiagen) utilise UNG in association with a PK digestion and resin‐column DNA purification. We therefore evaluated the effect of adding UNG to our improved protocol and also compared these results with those obtained with the Qiagen GeneRead system (Figure 3a). Incorporation of UNG, which included additional incubation of samples for 1 hr at 50°C, unexpectedly improved yield obtained from our improved protocol (Figure 3b) but had no effect on the purity (Figure 3c) or DNA fragment length (Figure 3d,e) of the obtained DNA. This improved yield was significantly greater than that produced by the Qiagen GeneRead system (Figure 3b) and this commercial system did not demonstrate improvements in DNA fragment length or purity. Importantly, there was no difference in the performance of the three methods in the PCR reaction and in all cases ample amounts of the 400‐bp fragments from the *HAND2* gene were readily amplified (Figure 3f).

**Figure 4:**
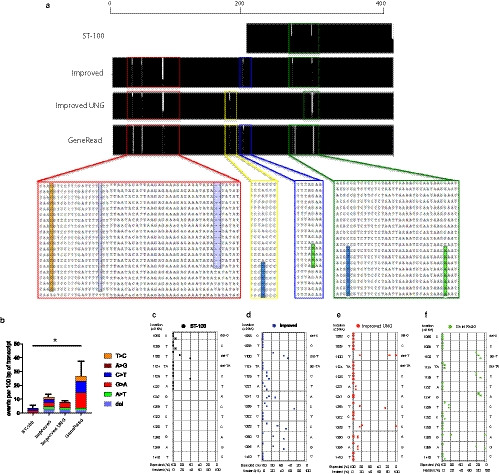
Fidelity of amplicons determined by Sanger sequencing. (a) Aligned sequence of 20 subcloned amplicons from each method, showing loci of altered bases at several discrete loci. DNA from the ST‐100 method was only able to produce 200‐bp‐length amplicons. (b) The mean number of variations from the expected Ensembl human sequence per 100 bp. Error bars: mean and SEM. *p < .05. (c‐f) Percentage of subcloned transcripts showing expected or aberrant identities at each locus. Each dot represents a single technical replicate, each of 20 clones

Formalin fixation leads to corruption of sequence as well as severing of DNA (Williams *et al.,* 1999; Do, 2012). We evaluated the extent of this sequence corruption, comparing our initial protocol with the improved protocol, with and without the addition of UNG. We also evaluated sequence in the DNA obtained using the commercial Qiagen GeneRead system. Bacterial sub‐cloning was performed to identify the frequencies of different sequence variations present in the PCR template pool. Sanger sequencing of 20 individual bacterial colonies, each from three different PCR reactions, was carried out for each of the protocols (Figure 4a). The initial ST‐100 protocol (involving heating to 100°C) provided 200‐bp amplicons. The improved protocol with and without UNG, and also the Qiagen GeneRead system, yielded 400‐bp amplicons suitable for sequence analysis. There was no unfixed DNA sample directly relating to the fixed pulmonary artery and so it was not possible to know the original/true DNA sequence of the *HAND2* gene in the sample. To overcome this limitation, we sought to identify differences between different methods in comparison with the Ensembl reference assembly of the Human Genome. For all methods, the expected Ensembl genomic sequence was discernible within the three experimental replicates, each consisting of 20 sub‐cloned transcripts. Overall analysis indicated that deviations appeared limited to specific loci (Figure 4a). Deleted bases were rare findings (Figure 4b). The ST‐100, the improved protocol and the improved protocol with UNG produced fewer than 0.6 variations per 100 bases of transcript. In contrast, the Qiagen GeneRead system produced three times as many variations (Figure 4b). More importantly, using bacterial sub‐cloning it was possible to ascertain whether the frequency of aberrations within amplicons would lead to a false conclusion, for example, if 50% of subclones or 100% of subclones were aberrant, the patterns might suggest heterozygosity or homozygosity for the variant. Using the ST‐100 protocol there was clear indication of the expected Ensembl base at most loci except for a deletion of T at position 1,100, and C indicated instead of T at position 1,126 in one of the bacterial cloning replicates. Although heterozygosity for a single base deletion seems unlikely, the C insertion is plausible and could be misrepresented. Similarly, with the improved protocol, there were several positions with variation: 1100delT, 1259T > C, 1328T > C, 1350G > A, 1395A > G, but the canonical genomic sequence was still recognised within other transcripts at sufficient levels to exclude heterozygosity or homozygosity of the variant. These loci still exhibited variation when UNG was added to our improved protocols or when the GeneRead kit was used. However, based on their incidence of approximately 50%, the 1259T > C and 1328T > C variants could now be considered plausible polymorphisms (Figure 4e,f). Taken together these data indicate that the use of Chelex resin and autoclaving to obtain DNA from formalin‐fixed samples provides significant advantages over other methods in terms of both yield and efficacy as a PCR template. Importantly, the maximal length of PCR amplicons produced relate well to the fragment size distribution of the genomic DNA template, indicating optimal PCR. Conversely, although the use of UNG may remove deamination artefacts, it also appears capable of introducing confusing sequence changes. Thus, our studies do not support the use of UNG, as bacterial sub‐cloning of amplicons prepared without UNG appear to provide greater guidance as to the genomic DNA sequence.

### 3.3 Comparison of next generation exome sequencing results from formalin‐fixed tissue and lymphocyte‐derived DNA

**Figure 5:**
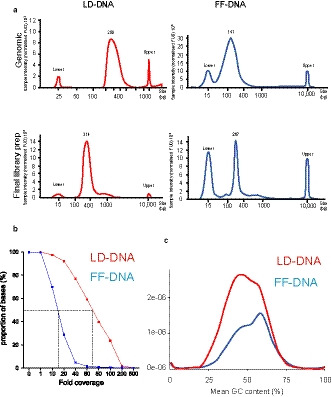
Library preparation for next generation sequencing. (a) Lymphocyte‐derived (LD) or formalin‐fixed (FF) DNA fragment size distribution in original genomic DNA and final library preparations by Tape Station analysis (lower and upper are internal standards). (b) Depth of read and (c) mean GC content for DNA samples from BAM QC analysis

**Figure 6:**
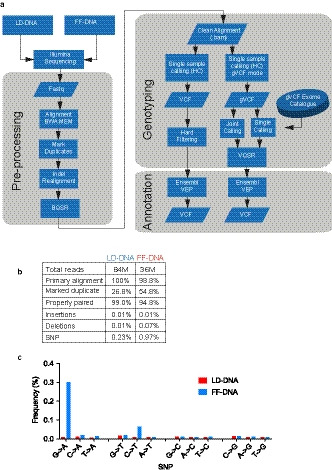
Bioinformatic processing pipeline. (a) Lymphocyte‐derived (LD) or formalin‐fixed (FF) DNA was sequenced using Illumina NextSeq500. Exomes were analysed using GATK 3.4 pipeline (see Methods for full details). Sequence data was aligned with BWA‐mem and duplicate reads marked and rearrangements performed for insertions and deletions. Base quality score recalibration (BQSR) was carried out to produce BAM files ready for variant calling through HaplotypeCaller (HC). Single sample calling was performed to assess the effect of hard filtering and both pair and single calling with an exome catalogue to assess the effect of joint calling. Ensembl Variant Effect predictor (VEP) was used to annotate transcripts as variant call files (VCF). (b) Total reads per sample and characteristics of BAM files used for Haplotype Caller. (c) Frequency of single nucleotide polymorphisms reported in (b)

Finally, we asked how optimally extracted genomic DNA from formalin‐fixed tissue would perform as template for next generation whole exome sequencing. To do this, we made use of a myocardial tissue sample that had been stored in formalin for 2 years and were able to compare this FF‐DNA with lymphocyte‐derived DNA (LD‐DNA) from the same individual, which had been sequenced as part of another study. Extraction of DNA from the myocardial sample was performed using the optimised protocol without UNG (Figure 3a), both because of uncertainty as to what distortions were resulting for the use of this enzyme and also to establish the performance of bioinformatic processing in identifying artefactual changes. The FF‐DNA obtained was already highly fragmented, with a mean fragment length of 141 bp (Figure 6a), and further fragmentation was not required for NGS. Library preparation was therefore performed with the TrueSeq Exome kit (Illumina). The LD‐DNA library was prepared using TrueSeq Rapid Exome kit (Illumina), an identical product that incorporated transposonase‐based fragmentation to yield median insert lengths of 150 bp (Figure 6a). Both the TrueSeq Exome and the TrueSeq Rapid Exome kits utilised the same library preparation reagents and Hg19 bed file. In both cases, NGS was carried out in a conventional way using an Illumina NextSeq (Illumina). There was equal loading of library preparation, collecting 80 million 75 base pair end reads, divided across four flow cells. Global analysis of read coverage indicated less depth of reads in the FF‐DNA sample compared with LD‐DNA. Hence median depth of coverage in the LD‐sample was greater than 75‐fold, whereas median depth of coverage in FF‐DNA was approximately 15‐fold (Figure 5b). Despite this reduction in depth of coverage, the overall GC content profiles were similar (Figure 5c), suggesting that the reduction in read depth was distributed evenly across the exomes in FF‐DNA.

Data from NGS were analysed using a bioinformatic analysis pipeline based on GATK Best Practice 3.4 (Figure 6a). Pre‐processing and analysis of reads to generate sequence variants did not involve significant options to alter until use of HaplotypeCaller.

Preliminary analysis of the BAM files obtained after pre‐processing (Figure 6b) showed that the FF‐DNA yielded less than half the number of total reads compared with LD‐DNA, and there were twice as many reads marked as duplicate. This and the reduction in fold coverage are in keeping with the degree of fragmentation present in the FF‐DNA, limiting the amount of functional template available for sequencing in the flow cells. Despite this, the quality of the read mapping appeared high, with 98.8% of reads finding primary alignment and 94% of paired reads being properly paired. During indel realignment there were more deletions noted in the FF‐DNA sample and, as expected, an increase in the frequency of apparent single nucleotide polymorphisms (SNPs) (Figure 6b). Closer inspection indicated the SNPs were in keeping with formalin corruption effects, with G > A and C > T predominating (Figure 6c).

### 3.4 Bioinformatic analysis impacts on sensitivity and specificity of variant calling

Once sequencing data have been pre‐processed they are ready to be compared with the human genomic scaffold, using HaplotypeCaller to identify genomic variants. Further filtering is required to remove artefacts due to erroneous reads. There are several ways to do this within the GATK toolkit and we were able to compare the performance of these on FF‐DNA, using LD‐DNA results as a ‘gold standard’. Ideally, if FF‐DNA were as reliable as LD‐DNA, there would be complete matching of findings from both samples and therefore 100% specificity (no false positive or spurious findings) and 100% sensitivity (no false negative or missed findings). The first analysis compared variants produced by single sample calling with hard filtering (hard filter settings: QualByDepth [QD] 2.0; FisherStrand [FS] 60.0; RMSMappingQuality [MQ] 40.0; MappingQualityRankSumTest [MQRankSum] −12.5; ReadPosRankSumTest [ReadPosRankSum] −8.0; StrandOddsRatio [SOR] 3.0). Hard filtering led to recognition of 13,079 variants in the LD‐DNA sample but only 5,239 variants in the FF‐DNA, indicating a sensitivity and specificity of 13.0% and 32.5%, respectively, for detection of variants using FF‐DNA. This low sensitivity coupled with low specificity indicated hard filtering would be of limited use for prima facie detection of DNA variants. An alternative and highly effective approach is to use a catalogue of exomes (216 in this case) to provide statistical support for variant quality score recalibration (VQSR). We initially applied this method to LD‐DNA and FF‐DNA at the same time (i.e. joint calling of both samples together), using the 216‐exome catalogue. As HaplotypeCaller is provided with the LD‐DNA ‘gold standard’ data at the same time as the FF‐DNA test set, this should produce the most optimal level of filtering possible. A total of 136,854 variants were identified in the LD‐DNA sample and 110,741 in the FF‐DNA. Compared with hard calling, this produced a 10‐fold increase in variants identified in LD‐DNA and 20‐fold increase in variants identified in FF‐DNA. This translated to a maximal sensitivity and specificity of 76.3% and 94.2%, respectively, for identification of variants using FF‐DNA.

**Figure 7:**
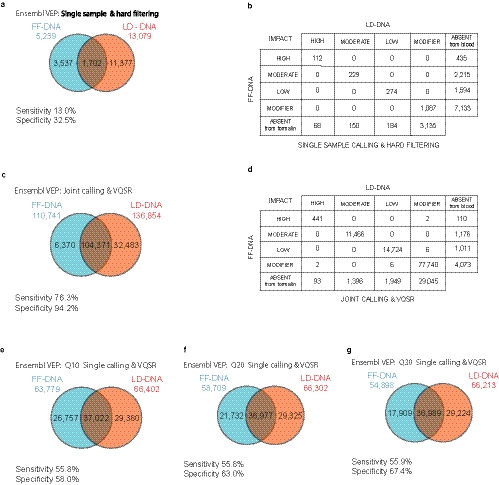
Comparison of GATK variant calling methods. (a) Venn diagram showing frequency of variants reported by Ensembl VEP after GATK pipeline with hard filtering. (b) Table comparing Ensembl VEP classification of variants between DNA samples in (a). (c) Venn diagram showing the effect of joint calling FF‐DNA and LD‐DNA with 216 exome catalogue. (d) Table comparing Ensembl VEP classification of variants between DNA samples in (c). (e‐g) Effect of increasing Phred Score on separate joint calling of FF‐DNA and LD‐DNA using 216 exome catalogue

We then asked whether subtle disturbances in the classification of variants during downstream analysis, e.g. when using Ensembl Variant Effect Predictor (Ensembl VEP), might occur in the FF‐DNA sample. Reassuringly, there were no differences when we compared the impact classifications of variants from LD and FF DNA samples (Figure 7b,d).

Presenting the HaplotypeCaller with FF‐DNA and LD‐DNA data at the same time allowed the best possible identification of variants within the FF‐DNA sample, as the ‘correct’ results were available from the LD‐DNA dataset to inform analysis of the FF‐DNA data directly. However, in analysing archival hearts or historical tissue samples the LD‐DNA would not be available and FF‐DNA would be analysed solely in comparison with the exome catalogue. We therefore compared the results obtained by calling each DNA sample separately using VQSR, together with the 216‐exome catalogue.

We also took the opportunity to examine the effect of filtering based on the quality of original sequencing reads, indicated by the Phred Score (Q), which can be used to identify reads that have a high probability of exhibiting base calling errors. For example, a score of Q30 describes a base with an error probability of 1 in 1,000, or likely to be 99.9% accurate, whereas the Q score of 10 (Q10) equates to a likely error rate of 1 in 10, or 90% accuracy. Normally Q30 is used, as high‐quality genomic DNA is unlikely to contain many errors. However, in our case we wondered whether accepting a lower threshold for errors might maintain transcripts with errors in different places (as might be expected as a response to formalin fixation), providing more depth of accurate base sequence overall. Using a Phred score of 10, separate VQSR analysis of lymphocyte DNA produced 66,402 variants, whereas FF DNA produced 67,519 variants. The sensitivity and specificity were reduced to 55.8% and 58%, respectively. The increased stringency achieved by increasing Phred scores to Q20 or Q30 had no effect on the number of variants reported in LD‐DNA but markedly reduced the number of variants reported from FF‐DNA to 58,709 at Q20 and 54,898 at Q30. This did not change the capability of the VQSR correctly to identify variants (sensitivity remained at 55.8%–55.9%) but efficiently reduced the number of false variants and increased specificity from 58% to 63% and 67.4%, respectively. Taken together, these analyses indicate that group calling with the GATK pipeline is an effective method of filtering variants from FF‐DNA and that there is additional benefit in using higher levels of stringency with FF‐DNA.

## 4 DISCUSSION

In this study, we have optimised the extraction of DNA obtained from overly formalin‐fixed human tissue and evaluated its performance as a template for chain termination (Sanger) sequencing and NGS. Proteinase K is a preferred reagent to digest tissue and liberate DNA for such molecular biology studies. However, the presence of residual formalin within tissue that might inactivate the enzyme has been a concern and this has led to the production of protocols with extended washing and repeated addition of fresh reagent (Legrand *et al.,* 2001; Cao *et al.,* 2003; Shi *et al.,* 2004; Pandey *et al.,* 2014; Idris, 2015). This increases the time needed to extract DNA and also makes the process expensive. Our studies have shown that extensive washing is not required and that halving the amount of Proteinase K used in published protocols does not affect the collection of high‐quality DNA. It is likely that further reductions will be possible. Although our studies are focused on overly formalin‐fixed and sub‐optimal tissue samples, it is likely our protocols will be of value in other less extremely corrupted formalin‐fixed samples. Following digestion, the extraction of DNA was enhanced both by autoclaving and by addition of the Chelex resin. Autoclaving provides more energy to separate covalent bonds (Shi *et al.,* 2004) but the mechanism of action for Chelex is unclear. Chelex may sequestrate metal ions and protect from DNAses (Walsh *et al.,* 1991) or, alternatively, it may bind to and protect denatured single strand template DNA, shielding it from fragments of complementary DNA that would normally interfere with the PCR reaction (Dietrich *et al.,* 2013). The reduction in yield but improvement in PCR performance when both were used together may reflect greater stripping of contaminating fragments to reveal cleaner, but less abundant high‐quality template. Thus, the efficacy of a DNA extraction protocol is reflected less by the yield or fragment length of DNA collected and more by the length of amplicons, which closely match the fragment length of the template genomic DNA. It is also possible that quantitation of DNA extracted from formalin‐fixed tissues may be less accurately measured by processes such as spectrophotometry because of variable fragmentation.

Bacterial sub‐cloning allowed us to identify clearly the range of transcript sequences and thus accurately determine the fidelity of sequence in the PCR template. Although the fragmentation of DNA produced by formalin exposure cannot be reversed, it has been suggested that the corruption of DNA by deamination to produce uracil residues can be overcome by treatment with UNG (Do, 2012). In vivo*,* UNG excises deaminated bases, which are then enzymatically repaired in relation to the opposite strand. In vitro, the template is simply cleaved, preventing the PCR from extending along that template. Surprisingly, changes in base sequence within the transcript pool appear after UNG usage, as revealed by bacterial subcloning, especially in DNA extracted using the Qiagen GeneRead system. This phenomenon has not been described previously, but this bias towards aberrant base calls may reflect the limited amount of DNA purified on the spin column tubes. Further studies are warranted to confirm and explore why this occurs. The Q5 polymerase used in the experiments is a high‐fidelity reagent with extremely low error rates and is unlikely to be responsible for the observed sequence errors. However, it is clear that the results obtained using our improved protocol of DNA extraction, with and without UNG, are very similar. Thus until the biology of UNG is more fully understood, there is no advantage from its use.

NGS is now a relatively cheap and rapid method of reading nucleic acids by generating millions of short overlapping sequences and it should be ideally suited to fragmented DNA, as shearing of DNA into short fragments is a requirement of the process. However, the un‐corrupted DNA template obtained from FF tissues is diluted by other template fragments of inherent poor quality, leading to a reduced number of successful reads and more duplicates. Filtering high‐quality reads from corrupted reads is essential and highly dependent on bioinformatic processes. The joint calling approach using VQSR and an exome catalogue is extremely useful. In this study, we have shown that although sensitivity cannot be improved by joint calling and variant quality score recalibration, changing the stringency of read quality by increasing Phred score filtering to Q30 has a major effect on the specificity.

Whereas for population‐based studies, some degree of error due to formalin corruption can be tolerated and differences between groups can still be shown to be statistically different, this is not appropriate for analysis of individuals. With maximal specificity of 67% and sensitivity of 56%, there is a major risk of missing a disease‐causing variant or, conversely, suggesting one in error. Historically, formalin‐fixed archival hearts have been used to investigate genes causing congenital heart malformations. For example, the Leipzig collection of malformed hearts comprises 292 formalin‐fixed human hearts collected between 1954 and 1982 (Craatz *et al.,* 2002) and has been used for genetic analysis. For example, analysis of hearts within the collection suggested that a mutation in *HAND1* (A126fs) might cause hypoplastic left heart syndrome (Reamon‐Buettner *et al.,* 2008). Although the authors took care to determine whether this was a true finding, in retrospect it seems likely that it was an artefact of formalin exposure and unlikely that the variant was responsible for the malformation in the archived hearts. Notably, their study suggested that many hearts with different forms of hypoplastic left heart syndrome had the same A126fs variant in *HAND1*, and their findings have not been confirmed by subsequent genomic studies (Esposito *et al.,* 2011) or animal modelling of the variant y (Firulli *et al.,* 2017).

Our study has shown that it is possible to extract DNA from formalin‐fixed tissues for genomic studies involving Sanger sequencing and NGS. Importantly, careful bioinformatic processing can reduce false negative findings, but approximately half of all variants will not be detected due to damage to the genomic DNA by formalin. We would therefore recommend that formalin‐fixed tissue can be used as a confirmatory source, for example when the presence in several family members has already suggested a specific variant, but in isolation, DNA from formalin‐fixed tissues is not a reliable source of novel variants. Finally, we suggest the use of bacterial sub‐cloning and Sanger sequencing of PCR amplicons to confirm variants of possible clinical relevance.

## AUTHOR CONTRIBUTIONS

AA, AS, SA DJH and BC contributed to concept/design; AA, LE acquisition of data; AA, AS, LE, DJH, and BC data analysis/interpretation. AA, AS, LE, SA, DJH and BC were involved in drafting, critical revision and approval of the article.
